# The *Sporothrix schenckii* Gene Encoding for the Ribosomal Protein L6 Has Constitutive and Stable Expression and Works as an Endogenous Control in Gene Expression Analysis

**DOI:** 10.3389/fmicb.2017.01676

**Published:** 2017-09-01

**Authors:** Elías Trujillo-Esquivel, José A. Martínez-Álvarez, Diana M. Clavijo-Giraldo, Nahúm V. Hernández, Alberto Flores-Martínez, Patricia Ponce-Noyola, Héctor M. Mora-Montes

**Affiliations:** División de Ciencias Naturales y Exactas, Departamento de Biología, Universidad de Guanajuato Guanajuato, Mexico

**Keywords:** *Sporothrix schenckii*, gene expression, protein glycosylation, sporotrichosis, RNA, glucosidase, mannosidase, dimorphism

## Abstract

*Sporothrix schenckii* is one of the causative agents of sporotrichosis, a worldwide-distributed mycosis that affects humans and other mammals. The interest in basic and clinical features of this organism has significantly increased in the last years, yet little progress in molecular aspects has been reported. Gene expression analysis is a set of powerful tools that helps to assess the cell response to changes in the extracellular environment, the genetic networks controlling metabolic pathways, and the adaptation to different growth conditions. Most of the quantitative methodologies used nowadays require data normalization, and this is achieved measuring the expression of endogenous control genes. Reference genes, whose expression is assumed to suffer minimal changes regardless the cell morphology, the stage of the cell cycle or the presence of harsh extracellular conditions are commonly used as controls in Northern blotting assays, microarrays, and semi-quantitative or quantitative RT-PCR. Since the biology of the organisms is usually species specific, it is difficult to find a reliable group of universal genes that can be used as controls for data normalization in experiments addressing the gene expression, regardless the taxonomic classification of the organism under study. Here, we compared the transcriptional stability of the genes encoding for elongation factor 1A, Tfc1, a protein involved in transcription initiation on Pol III promoters, ribosomal protein L6, histone H2A, β-actin, β-tubulin, glyceraldehyde 3-phosphate dehydrogenase, UAF30, the upstream activating factor 30, and the transcription initiation factor TFIID subunit 10, during the fungal growth in different culture media and cell morphologies. Our results indicated that only the gene encoding for the ribosomal protein L6 showed a stable and constant expression. Furthermore, it displayed not transcriptional changes when *S. schenckii* infected larvae of *Galleria mellonella* or interacted with immune cells. Therefore, this gene could be used as control for data normalization in expression assays. As a proof of concept, this gene was used to assess the expression of genes encoding for glycosidases involved in the protein *N*-linked glycosylation pathway, a histidine kinase whose expression is regulated during the fungal dimorphism, and a glycosidase that participates in sucrose assimilation.

## Introduction

Sporotrichosis is a subacute or chronic infection caused by members of the *Sporothrix schenckii* complex, a group of dimorphic fungi with worldwide distribution (Lopes-Bezerra et al., 2006; Lopez-Romero et al., 2011; Chakrabarti et al., 2015). Although most of the sporotrichosis cases may be controlled with the recommended antifungal strategies, it has been recently considered as an emerging health problem due to the epidemic outbreaks in Brazil, associated to cats, and the high mortality and morbidity rates in immunocompromised patients (Lopez-Romero et al., 2011; Rodrigues et al., 2013, 2016). Despite its increased importance in both the basic research and the medical mycology field, the publication records dealing with these organisms and the infection are extremely limited, with less than 3000 research papers found in repository databases such as PubMed from the National Center for Biotechnology Information, since the first report of the disease by Schenk more than a century ago (Mora-Montes et al., 2015). The lack of interest in this field might be explained, in part, by the limited repertoire of molecular tools to facilitate the study of these organisms (Mora-Montes et al., 2015). The first approach to assess the relevance of genes and molecular pathways in *S. schenckii* physiology and virulence was the use of mutants generated by exposure to UV light (Torres-Guerrero and Arenas-Lopez, 1998; Romero-Martinez et al., 2000). Later, a protocol to generate *S. schenckii* insertional mutants based on *Agrobacterium tumefaciens* transformation was reported (Zhang et al., 2011), the RNA interference approach was standardized for the study of *S. schenckii* calcium/calmodulin kinase I (Rodriguez-Caban et al., 2011); and more recently, the genome sequencing of *S. schenckii sensu stricto*, *S. brasiliensis*, *S. globosa*, and *S. pallida* were reported (Teixeira et al., 2014; D’Alessandro et al., 2016; Huang et al., 2016).

The gene expression analysis is a set of powerful tools that helps to assess the cell response to changes in the extracellular environment, the genetic networks controlling metabolic pathways, and the adaptation to different growth conditions or the presence of extracellular insults. Most of the quantitative methodologies used nowadays require data normalization, and this is achieved running experiments in parallel to measure the expression of endogenous control genes. Reference genes, whose expression is assumed to suffer minimal changes regardless the cell morphology, the stage of the cell cycle or the presence of harsh extracellular conditions (Velculescu et al., 1999; Cusick et al., 2014), are commonly used as controls in Northern blotting assays, microarrays, and semi-quantitative or quantitative RT-PCR (RT-qPCR). Since the biology of the organisms is usually species specific, it is difficult to find a reliable group of universal genes that can be used as controls for data normalization in experiments addressing the gene expression, regardless the taxonomic classification of the organism under study. In several works, it is clear that the genes routinely used as controls for data normalization, such as actin, the ribosomal subunit 18S, β-tubulin, and others; are not always expressed constitutively in all the studied organisms (Zhong and Simons, 1999; Hamalainen et al., 2001; Selvey et al., 2001; Deindl et al., 2002; Glare et al., 2002; Artico et al., 2010). In fact, a gene with constitutive a stable expression in an organism displays different gene expression in others. Thus, it has been suggested that the best way to normalize expression data is the establishment of a panel of multiple reference genes, specific for each organism (Vandesompele et al., 2002).

In this work, we aimed to find genes whose expression is stable and constitutive under different growth conditions of *S. schenckii*. Our data indicated that the gene encoding for the ribosomal protein L6 showed a stable and constant expression in different growth conditions and cell morphologies. As a proof of concept, the identified gene was used to assess the expression of genes encoding for glycosidases involved in the protein *N*-linked glycosylation pathway, a histidine kinase whose expression is regulated during the fungal dimorphism, and a glycosidase that participates in sucrose assimilation.

## Materials and Methods

### Strains and Culturing Conditions

The following clinical isolates of *S. schenckii sensu stricto* (kindly donated by Prof. Leila M. Lópes-Bezerra, University of Rio de Janeiro State, Brazil) were used in this study: 1099-18 (ATCC MYA-4821) (Castro et al., 2013), Ss39, Ss47, and SS-B02 (Fernandes et al., 2013). Unless otherwise indicated, strain 1099-18 (ATCC MYA-4821) was the organism used throughout the study. Cells were grown and maintained at 28°C in YPD medium (1% [w/v] yeast extract, 2% [w/v] gelatin peptone, 3% [w/v] dextrose), added with 2% (w/v) agar when required. The mycelial morphology was obtained as previously described (Mora-Montes et al., 2010), with minimal modifications. Briefly, 5 mL of overnight-grown cells at 28°C in YPD broth, pH 4.5, were inoculated into 50 mL fresh medium, and incubated at 28°C and shaking at 200 rpm for 36 h. Then, mycelia were harvested by filtering, using a vacuum system and a 5-μm nylon membrane (Monodur^®^), and washed six times with sterile cold water during the process. Cells were frozen in liquid nitrogen and kept at -70°C until used. Yeast-like cells were obtained as follows: a 5-mL aliquot of 24 h-grown cells at 37°C in YPD broth, pH 7.8, was inoculated into 50 mL of fresh YPD broth, pH 7.8, and incubated at 37°C with constant shaking at 120 rpm for 4–6 days. Under these conditions, nearly 100% cells displayed a yeast-like morphology. Cells were pelleted by centrifuging at 5000 × *g* for 5 min at 4°C, washed three times with deionized cold water, and placed in liquid nitrogen until used.

To grow fungal cells in several carbon and nitrogen sources, cells were cultured in 5 mL of YPD broth, pH 7.8, at 37°C and shaking (120 rpm) for 3–4 days. Then inoculated in 50 mL of each tested broth and incubated at 28°C and 200 rpm during 48 h. Finally, cells were collected by centrifuging at 5000 × *g* for 5 min at 4°C before RNA extraction. Media tested were brain–heart infusion (BHI, Oxoid), PDB (0.4% [w/v] potato extract and 2% [w/v] dextrose) and Vogel (1.5% [w/v] sucrose and Vogel 1X from stock solution 50X). Vogel stock solution 50X included: 20% (w/v) sodium citrate⋅5_1/2_ H_2_O, 33.33% (w/v) K_2_HPO_4_, 13.33% (w/v) NH_4_NO_4_, 1.33% (w/v) MgSO_4_⋅7H_2_O, 0.66% (w/v) CaCl_2_⋅2H_2_O, 0.66% (v/v) trace element solution (5% [w/v] citric acid⋅H_2_O, 5% [w/v] ZnSO_4_⋅7H_2_O, 1% [w/v] Fe(NH_4_)_2_(SO_4_)_2_⋅6H_2_O, 0.25% [w/v] CuSO_4_⋅5H_2_O, 0.05% [w/v] MnSO_4_⋅H_2_O, 0.05% [w/v] H_3_BO_4_, 0.05% [w/v] Na_2_MoO_4_⋅2H_2_O, and 1% [v/v] chloroform), 0.33% (v/v) biotine at 0.01% (w/v), and 0.2% (v/v) chloroform. For induction of β-fructofuranosidase activity, conidia were inoculated in medium contained 0.67% (w/v) yeast nitrogen base and 3% (w/v) sucrose (Sigma), and incubated at 28°C for 72 h and 120 rpm (Lutfiyya and Johnston, 1996). As control, cells were inoculated in 0.67% (w/v) yeast nitrogen base and 3% (w/v) glucose, and incubated under the same conditions.

### Interaction of *S. schenckii* with *Galleria mellonella* Larvae

Killing assays of *Galleria mellonella* larvae were conducted as described (Clavijo-Giraldo et al., 2016). Groups containing 10 larvae of at least 1 cm length and uniform color were inoculated in the last left proleg with 10 μL of a solution containing 1 × 10^5^ yeast/μL, using a Hamilton syringe (701N, 26’s gauge, 10 μL capacity). *Sporothrix* yeast-like cells were obtained by growing cells in YPD broth, pH 7.8, as described above. Animal groups were kept at 37°C and decapitated either at days 1, 5, 10, or 15 post-inoculation. Fungal cells were retrieved from the hemolymph by washing twice with cold deionized water, and frozen at -70°C for RNA extraction.

### Ethics Statement

Universidad de Guanajuato, though the Ethics Committee, approved the use of human cells in this study (permission number 17082011). Only healthy adult volunteers took part of this study. Information of the study was disclosed and written informed consent was signed before blood samples were withdrawn.

### Human Peripheral Blood Mononuclear Cells-*Sporothrix schenckii* Interaction

Histopaque-1077 (Sigma) was added to whole human blood and peripheral blood mononuclear cells (PBMCs) were isolated using density centrifugation as described (Martinez-Alvarez et al., 2017). Cells were washed twice in sterile PBS and suspended in RPMI 1640 Dutch modification (Sigma). The PBMC-fungus interaction was performed in 96-well microplates, with a human cell:yeast-like cells ratio 5:1, in a total volume of 200 μL containing 5 × 10^5^ PBMCs. Plates were incubated at 37°C with 5% (v/v) CO_2_, and the content of 10 wells was removed either at 1, 6, or 24 h after the beginning of the cell–cell interaction. Human cells were disrupted by washing twice with cold deionized water, and fungal cells were saved and frozen at -70°C for RNA extraction.

### Interaction of *S. schenckii* with RAW 264.7 Macrophages

The RAW 264.7 (ATCC^®^ TIB-71^TM^) murine cell line was cultured in DMEM media (Sigma), supplemented with 10% fetal bovine serum, at 37°C and 5% (v/v) CO_2_. After reaching 90% confluence, cells were detached using trypsin (Sigma) in DMEM medium and sub-seeded into 6-well plates for further growth. Cells were harvested by trypsin detachment and counted to be adjusted at a cell suspension of 2 × 10^5^ cells/mL in DMEM medium. The macrophage:yeast-like cell ratio was set at 1:5, and interactions were incubated at 37°C under a 5% (v/v) CO_2_ atmosphere. The content of 10 wells was removed either at 1, 6, or 24 h after the beginning of the cell–cell interaction. Murine cells were disrupted by washing twice with cold deionized water, and fungal cells were frozen at -70°C for RNA extraction.

### Extraction of Nucleic Acids

Genomic DNA was isolated as reported (Robledo-Ortiz et al., 2012). Briefly, frozen cells in liquid nitrogen, were ground with a mortar and pistil, resuspended in 200 mM Tris-HCl, pH 8.5, 250 mM NaCl, 25 mM EDTA, 0.5% (w/v) SDS, 40 μg ml^-1^ RNase, and incubated 1 h at 37°C. Then, DNA was extracted with one volume of a phenol:chloroform (1:1) solution, the sample was centrifuged 10 min at 8,000 × *g* and 4°C, the supernatant saved, mixed with 0.5 volumes of isopropanol, and incubated overnight at -20°C to precipitate DNA. The nucleic acid was washed with 70% (v/v) ethanol, and kept at -20°C until used. Total RNA was isolated following a protocol previously standardized (Logemann et al., 1987). Nitrogen-frozen cells were ground with mortar and pistil, 100 mg were placed in Eppendorf tubes containing 8 M guanidine hydrochloride, 20 mM MES, and 20 mM EDTA, mixed thoroughly in vortex and centrifuged 15 min at 10,000 × *g* and 4°C. The supernatant was saved, mixed with two volumes of acid phenol:chloroform(1:1), vortexed 5 min and centrifuged for 10 min at 10000 × *g* and 4°C. The aqueous phase was saved, RNA precipitated with one volume of absolute isopropyl alcohol, incubated 30 min at room temperature, then pelleted by centrifuging 10 min at 10,000 × *g* and 4°C, washed once with DEPC-treated 2 M sodium acetate, pH 5.2, and then twice with 70% (v/v) ethanol. The pelleted material was resuspended and kept at -70°C until used.

### Synthesis and Purification of cDNA

The quantification of nucleic acids was performed by spectrophotometry, using a NanoDrop 2000 (Thermo Scientific). Reverse transcription reactions were performed in a Thermal Cycler T100 thermocycler (BIO-RAD), using Superscript II reverse transcriptase (Invitrogen), 40 μg total RNA as template and oligo(dT) primer (20 mer), following the manufacturer’s instructions. Then, cDNA was purified using a recent protocol developed by our group that involves RNA degradation and adsorption chromatography (Trujillo-Esquivel et al., 2016).

### Assay Performance

Calibration curves for each quantified amplicon were generated using the following dilutions of cDNA synthesized with oligo(dT) primer (20 mer) as follows: 1.28, 6.4, 32, 160, and 800 ng/μL. The reactions were processed as described below to determine the threshold cycle (Ct). Ct values and template concentrations were used to generated a calibration curve and from that the efficiency of amplification was calculated as previously reported using the linear dynamic range (Bustin et al., 2009).

### Analysis of Gene Expression by RT-qPCR

The RT-qPCR reactions were performed using a thermocycler StepOne plus (Life Technologies) and the SYBR Green PCR Master Mix (Life Technologies). The reaction contained in a final volume of 20 μL: master mix, 100 nM each primer set (see **Table 1**), and 1 μg purified cDNA. Reactions were run as follows: 1 cycle at 95°C 10 min and 40 cycles at 95°C 15 s and 60°C 1 min. The StepOne software V 2.2 (Life Technologies) was used to analyze, quantify and validate the RT-qPCR reactions, determining the Ct and dissociation curves. Relative quantification was determined in the same software by calculating 2^-ΔΔC_*t*_^ (Livak and Schmittgen, 2001).

**Table 1:** Oligonucleotide primers used in this study.

Encoding gene for	Sequence of the primer pair	Amplicon size from genomic DNA (bp)	Amplicon size from cDNA (bp)
18S ribosomal RNA (XR_001677158)^∗^	5′-CAGTACAAGATTCCCTCCTG-3′ and 5′-CCCTTCTTCTTGGACACAC-3′	233	233
Elongation factor 1A (XM_016731693)^∗^	5′-AAGACTCACATCAACGTCG-3′ and 5′-ACTTCCACAGAGCAATATCG-3′	814	226
Tfc1, a transcription initiator on Pol III promoters (XP_016585977)^∗^	5′-CACTACAGTCACGACATCC-3′ and 5′-AACATAGGCAAGACAGAAGG-3′	232	232
Ribosomal protein L6 (XP_016584434)^∗^	5′-ATTGCGACATCAGAGAAGG-3 and 5′-TCGACCTTCTTGATGTTGG-3′	303	224
Histone H2A (XP_016582554)^∗^	5′-TTCCAAGAACTCGCAGAC-3′ and 5′-TCAATTCCTCATCGTTCCG	339	251
β-Actin (XP_016586421)^∗^	5′-GGTATCATGATCGGTATGGG-3′ and 5′-CTGGGTCATCTTCTCACG-3′	240	240
β-Tubulin (XP_016589605)^∗^	5′-ACTCGTCGTACTTTGTTGAG-3′ and 5′-ACTCCATCTCGTCCATACC-3′	213	213
Glyceraldehyde 3-phosphate dehydrogenase (XP_016586602)^∗^	5′-ATCAAGGCCGCTATTAAGG-3′ and 5′-CTATCGACCTTGGCTACG-3′	236	236
Upstream activating factor 30 (XP_016588691)^∗^	5′-TTCCAAAAGCCCTTCAACC-3′ and 5′-GATATAGGTGGTTGCTGAGC-3′	229	229
Transcription initiation factor TFIID subunit 10 (XP_016584681)^∗^	5′-GATCATGCTACTGGTGGTG-3′ and 5′-CCTTGGTCATGTAGTAGTTGG-3′	259	187
β-Fructofuranosidase (XP_016588935)^∗^	5′-GTCTTCTGTCACCAACACCT-3′ and 5′-ACATTGTCTGGATCGTAACC-3′	180	180
Mannosyl-oligosaccharide glucosidase (XP_016592619)^∗^	5′-ATCGGCTACTTTTACGGC-3′ and 5′-AACCAGCTCTTGACAATGTC-3′	260	260
α-Glucosidase II (XM_016735056)^∗^	5′-CTCTGGACATTTCTTTCGTG-3′ and 5′-CCTTCTCCTTGGTGATGTC-3′	270	270
Mannosyl-oligosaccharide α-1,2-mannosidase (XM_016732698)^∗^	5′-GCGTCAACATTGGAAAGTC-3′ and 5′-GTATTCGTAGTAGGAGTCGC-3′	278	278
Hybrid histidine kinase DRK1 (JX416706)^∗^	5′-TCAATGCACAGCTTTTGGAG-3′ and 5′-GTGTTCATGCGGACCTTCTT-3′	202	202

^∗^*The accession code at the NCBI database (https://www.ncbi.nlm.nih.gov) is provided between brackets*.

### Quantification of Glycosidase Activity

The α-mannosidase and α-glucosidase activities were measured using the fluorogenic substrates 4-methylumbellyferyl-α-D-mannopyranoside (Sigma) and 4-methylumbellyferyl-α-D-glucopyranoside (Sigma), respectively. Cells were harvested by centrifugation, broken with glass beads in a FastPrep machine (Thermo Scientific), and the homogenate centrifuged at 21,000 × *g* and 4°C for 10 min. The supernatant was saved and used to quantify enzyme activity as reported (Mora-Montes et al., 2004; Robledo-Ortiz et al., 2012). Aliquots containing 100 μg protein were resuspended in 10 mM phosphate buffer, pH 7.0, in a total volume of 200 μL. Then, 40 μM of either 4-methylumbellyferyl-α-D-mannopyranoside or 4-methylumbellyferyl-α-D-glucopyranoside were added and the reaction incubated at 37°C for 30 min. The reaction was stopped by addition of 3.3 mL 50 mM glycine-NaOH, pH 11.0, and fluorescence of the released 4-methylumbellyferone (MU) was measured in a Perkin-Elmer LS-5B luminescence spectrofluorometer, with excitation and emission set at 350 nm and 440 nm, respectively. Total enzyme activity was expressed as nmoles of MU min^-1^ total protein^-1^. In assays to inhibit mannosyl-oligosaccharide glucosidase, 10 μM castanospermine (Sigma) was added prior the incubation step at 37°C (Lopes-Bezerra et al., 2015).

### Statistical Analysis

Statistical analysis was performed using GraphPad Prism 7 software. All experiments were performed three times in duplicate. Data represent cumulative results of all experiments performed and are showed as mean ± SD. The Mann–Whitney *U*-test or the one-way ANOVA were used to establish statistical significance, which was set at *P* < 0.05.

## Results

### Selection of Candidate Genes to Display a Constitutive and Stable Expression in *S. schenckii*

It has been described that the most suitable candidates for constitutive and stable expression are genes encoding for housekeeping functions, i.e., genes in charge of the basic tasks during cellular maintenance, such as those related to metabolism, molecular recycling or structural roles (Zhu et al., 2008; Cusick et al., 2014; Pathan et al., 2017). After a thorough revision of the literature, we selected the 10 most popular genes used in fungal cells systems as endogenous controls during gene expression analysis: the genes encoding for 18S ribosomal RNA (Fang and Bidochka, 2006; Li et al., 2012), elongation factor 1A (Fang and Bidochka, 2006; Son et al., 2013), Tfc1, a protein involved in transcription initiation on Pol III promoters (Teste et al., 2009), ribosomal protein L6 (Nowrousian et al., 2003; Cusick et al., 2014), histone H2A (Davé et al., 2006), β-actin (Nailis et al., 2006; Bohle et al., 2007; Cusick et al., 2014), β-tubulin (Yan and Liou, 2006; Cusick et al., 2014), glyceraldehyde 3-phosphate dehydrogenase (Panepinto et al., 2002; Tavanti et al., 2003; Fang and Bidochka, 2006), UAF30, the upstream activating factor 30 (Moss et al., 2007), and the transcription initiation factor TFIID subunit 10 (Teste et al., 2009). Most of these genes contain putative introns; therefore, some of the primer pairs for PCR were designed to align in regions containing at least one putative intron. This design was useful to assess the potential contamination of RNA samples with genomic DNA (see **Table 1**). All the putative genes were confirmed to be present in the genomic DNA preparations, and all of them showed expression, as cDNA was amplified by RT-PCR (data not shown).

### Variation of Gene Expression under Different Growth Conditions

**FIGURE 1:**
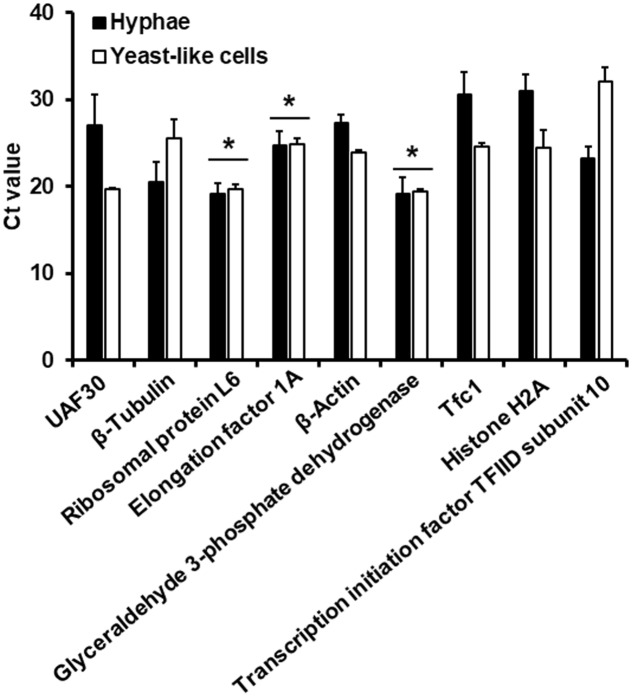
Ct values of genes with putative constitutive and stable expression in both *Sporothrix schenckii sensu stricto* hyphae and yeast-like cells. Cells were grown in YPD medium under controlled conditions to generated either hypha (closed bars) or yeast-like cells (open bars) (see Materials and Methods). Total RNA was extracted, cDNA synthesized with oligo(dT) primer (20 mer) and the gene expression was quantified by RT-qPCR using specific primers (see **Table 1**). Only the genes encoding the ribosomal protein L6, elongation factor 1A, and glyceraldehyde 3-phosphate dehydrogenase showed constant expression in both morphologies (^∗^*P* > 0.05).

Next, we compared the suitability to synthesize cDNA using either oligo(dT) primer (20 mer) or a specific reverse primer for the target gene. Upon RT-qPCR reactions, the Ct value for amplification of elongation factor 1A, using the specific reverse primer was 29.415 ± 1.273, whereas the Ct value of reactions with cDNA synthesized with oligo(dT) primer (20 mer) was 30.063 ± 0.998 (*P* > 0.05). Since no significant differences were observed, we decided to use the oligo(dT) primer (20 mer) during the transcriptase reverse reaction, to use the same cDNA preparation in the expression analysis of all the tested genes, thus minimizing variations. Since the 18S ribosomal RNA is not polyadenylated after transcription (Henras et al., 2015), this gene was discarded from the analysis. Synthesized cDNA using total RNA from conidia showed variations in the Ct values in all the analyzed genes, suggesting that RNA extraction from this morphology is not homogeneous across replicates. Since this phenomenon was not further investigated, we decided to eliminate conidia from our analysis. The efficiency of the amplification reactions was similar for all the primer pairs tested (**Table 2**). The comparison of Ct values for the nine genes with putative constitutive and stable expression in both yeast-like cells and hyphae are given in **Figure 1**. Three of the nine tested genes showed similar expression in both hyphae and yeast-like cells: those encoding the ribosomal protein L6, the elongation factor 1A and glyceraldehyde 3-phosphate dehydrogenase (**Figure 1**). The genes encoding for UAF30, β-actin, Tfc1 and histone H2A were significantly more expressed in yeast-like cells than in hyphae (**Figure 1**); while those encoding for β-tubulin and the transcription initiation factor TFIID subunit 10 showed more expression in hyphae than in yeast-like cells (**Figure 1**). Therefore, only the genes showing similar expression levels in both cell morphologies were further studied.

**Table 2:** Amplification reaction efficiency for the different amplicons analyzed in this study.

Encoding gene for	Amplification efficiency (%)	Correlation coefficient (*r^2^*)
Elongation factor 1A (XM_016731693)^∗^	94.3	0.9863
Tfc1, a transcription initiator on Pol III promoters (XP_016585977)^∗^	97.3	0.9936
Ribosomal protein L6 (XP_016584434)^∗^	98.8	0.9902
Histone H2A (XP_016582554)^∗^	93.2	0.9773
β-Actin (XP_016586421)^∗^	95.3	0.9873
β-Tubulin (XP_016589605)^∗^	92.5	0.9764
Glyceraldehyde 3-phosphate dehydrogenase (XP_016586602)^∗^	96.3	0.9843
Upstream activating factor 30 (XP_016588691)^∗^	98.7	0.9932
Transcription initiation factor TFIID subunit 10 (XP_016584681)^∗^	95.3	0.9902
β-Fructofuranosidase (XP_016588935)^∗^	98.4	0.9923
Mannosyl-oligosaccharide glucosidase (XP_016592619)^∗^	97.3	0.9963
α-Glucosidase II (XM_016735056)^∗^	93.4	0.9832
Mannosyl-oligosaccharide α-1,2-mannosidase (XM_016732698)^∗^	96.0	0.9897
Hybrid histidine kinase DRK1 (JX416706)^∗^	97.2	0.9956

^∗^*The accession code at the NCBI database (https://www.ncbi.nlm.nih.gov) is provided between brackets*.

**FIGURE 2:**
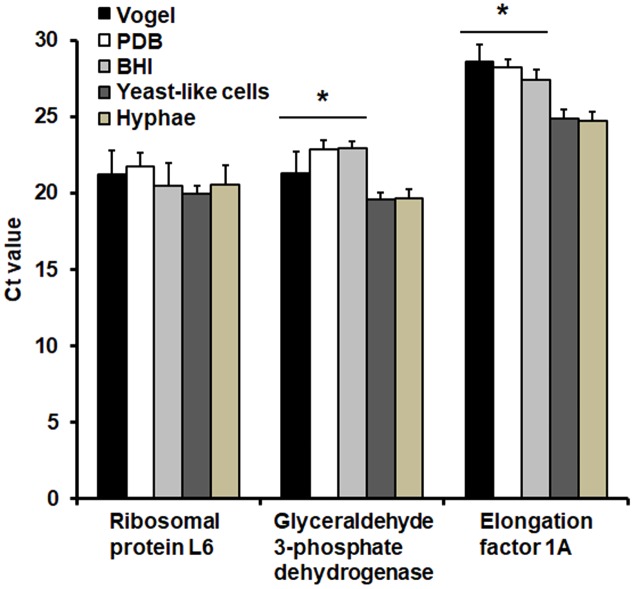
Ct values of genes with putative constitutive and stable expression in *S. schenckii sensu stricto* growing in different culture conditions. Cells were grown in either Vogel medium, potato-dextrose agar (PDB), brain–heart infusion (BHI), YPD, pH 4.5 (generates hyphae), or YPD, pH 7.8 (generates yeast-like cells) (see Materials and Methods). Total RNA was extracted, cDNA synthesized with oligo(dT) primer (20 mer) and the gene expression was quantified by RT-qPCR using specific primers (see **Table 1**). Only the gene encoding the ribosomal protein L6 showed constant expression in all the tested media. ^∗^*P* < 0.05 when compared to the expression level in hyphae and yeast-like cells.

Next, we tested the stability of the gene expression in different and popular culture media to grow molds, such as PDB and Vogel, and BHI, the most used culture medium to induce cell dimorphism in *S. schenckii* (Barros et al., 2011). In PDB, cells showed a morphology of long and thin filament cells; whereas in Vogel medium they were shorter filament cells and mixed with rounded yeast-like cells (data not shown). Cells displayed the classical yeast-like morphology when grown in BHI, but a small proportion of hyphae and conidia (less than 20% of total cell population) were also observed (data not shown). Cells obtained from these culture media, along with hyphae and yeast-like cells grown in YPD, were collected and used for total RNA extraction and RT-qPCR upon cDNA synthesis. When analyzed the expression of the gene encoding the ribosomal protein L6, no significant changes were observed in the Ct values across all the culture media tested (**Figure 2**). For the case of the genes encoding for the elongation factor 1A and glyceraldehyde 3-phosphate dehydrogenase, they displayed similar expression in yeast-like cells and hyphae obtained in YPD medium; but cells grown in either Vogel, PDB or BHI showed increased Ct values (**Figure 2**). Interestingly, for the gene encoding for the elongation factor 1A the expression levels in the three culture media (PDB, Vogel and BHI) were not significantly different (*P* = 0.1291). A similar situation was also observed with the gene encoding for glyceraldehyde 3-phosphate dehydrogenase (*P* = 0.0723). Therefore, only the gene encoding the ribosomal protein L6 showed stable and constitutive expression in the tested conditions.

### The *S. schenckii* Gene Encoding for the Ribosomal Protein L6 Does Not Show Differential Expression during Interaction with Host Cells

**FIGURE 3:**
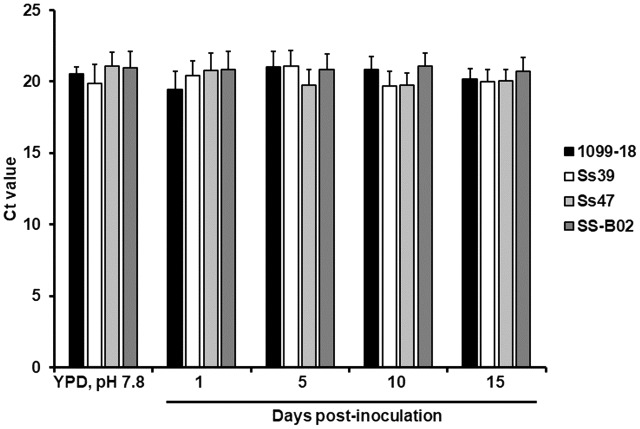
Ct values of the *S. schenckii sensu stricto* gene encoding for the ribosomal protein L6 upon interaction with *G. mellonella* larvae. *Sporothrix* yeast-like cells were obtained in YPD broth, pH 7.8, and used to inoculate animal groups containing 10 larvae. Animals were kept at 37°C and decapitated either at days 1, 5, 10, or 15 post-inoculation. Fungal cells were retrieved from hemolymph, total RNA was extracted, cDNA synthesized with oligo(dT) primer (20 mer) and the gene expression was quantified by RT-qPCR using specific primers (see **Table 1**). One-way ANOVA was used to establish statistical significance. *P*-value was for all the strains > 0.05.

Next, to further assess whether the expression of the gene encoding for the ribosomal protein L6 undergoes transcriptional regulation when fungal cells interact *in vivo* with host cells, we took advantage of the recently standardized model of sporotrichosis in larvae from *G. mellonella* (Clavijo-Giraldo et al., 2016), and infected the insect with yeast-like cells from the strain 1099-18 (ATCC MYA-4821). We followed the infection for 15 days and recovered fungal biomass at days 1, 5, 10, and 15 post-infection, to isolate RNA and determine the Ct values for the gene encoding for the ribosomal protein L6. No changes in the Ct values were observed in the selected points for fungal RNA isolation (**Figure 3**; *P* = 0.3234). Similar results were obtained with strains Ss39, Ss47 and SS-B02, which have different killing rates of larvae (Clavijo-Giraldo et al., 2016) (**Figure 3**).

**FIGURE 4:**
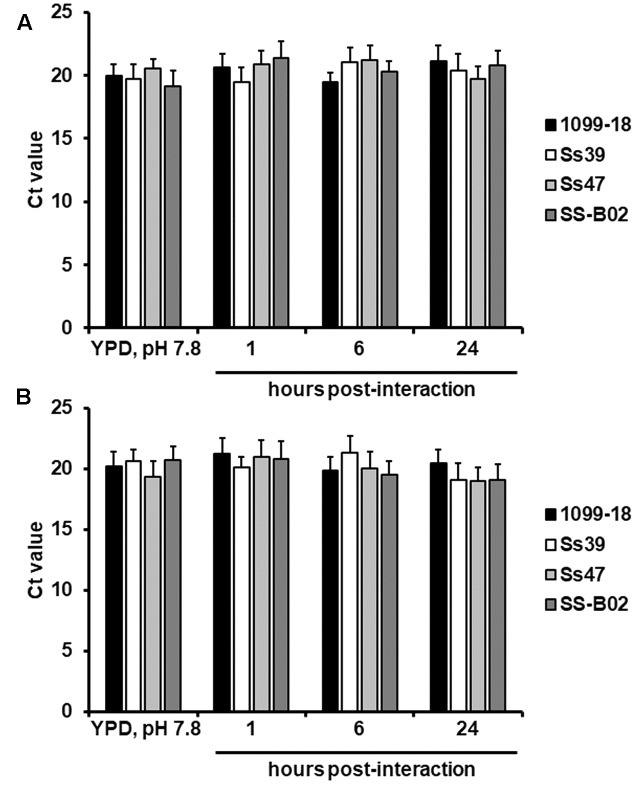
Ct values of the *S. schenckii sensu stricto* gene encoding for the ribosomal protein L6 upon interaction with human PBMCs and the RAW 264.7 (ATCC^®^ TIB-71^TM^) murine cell line. *Sporothrix* yeast-like cells were obtained in YPD broth, pH 7.8, and used to interact with either human PBMCs **(A)** of RAW 264.7 (ATCC^®^ TIB-71^TM^) murine cell line **(B)**. Fungal cells were retrieved after 1, 6, or 24 h interaction with the immune cells, total RNA was extracted, cDNA synthesized with oligo(dT) primer (20 mer) and the gene expression was quantified by RT-qPCR using specific primers (see **Table 1**). One-way ANOVA was used to establish statistical significance. *P*-value was for all the strains > 0.05.

To further expand our observations, we set *ex vivo* interactions between yeast-like cells of strain 1099-18 (ATCC MYA-4821) and human PBMCs, immune cells that are likely to participate in the establishment of a protective anti-*Sporothrix* immune response (Martinez-Alvarez et al., 2017). We retrieved fungal cells after 1 h (early stage) 6 h (intermediate stage) or 24 h (late stage) of interaction, and the Ct values of the gene encoding for the ribosomal protein L6 were not significantly different to those observed in cells grown in YPD, pH 7.8 (**Figure 4A**; *P* = 0.1872). Again, this was independent of the strain tested, as similar results were obtained when strains Ss39, Ss47, or SS-B02 were used (**Figure 4A**). Finally, when a similar approach was used to analyze the expression of the gene encoding for the ribosomal protein L6 during interaction with the murine macrophages RAW 264.7 (ATCC^®^ TIB-71^TM^), we could not find differences in its expression at 1 h, 6 h, or 24 h of interaction, or when different strains were used (**Figure 4B**). Therefore, expression of the gene encoding for the ribosomal protein L6 is likely to be stable and constant during *in vivo* and *ex vivo* interactions of *S. schenckii* with host cells.

### Validation of the Gene Encoding for the Ribosomal Protein L6 as a Useful Control for Data Normalization in RT-qPCR

**FIGURE 5:**
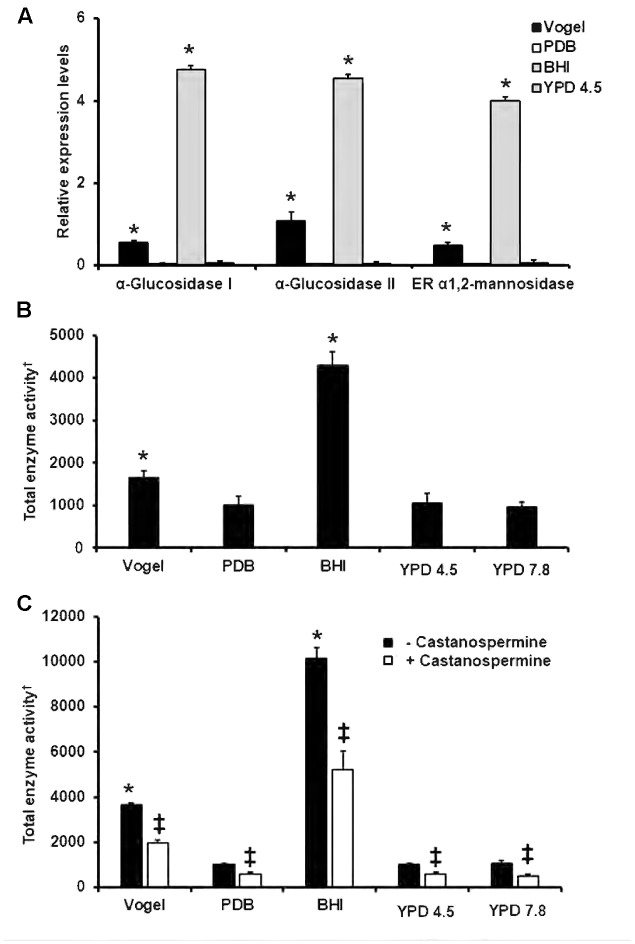
Gene expression and enzyme activity of endoplasmic reticulum (ER) α-glycosidases in different culture media. In **(A)**, gene expression of ER glycosidases was determined and normalized using the gene encoding for the ribosomal protein L6 as control, and the fungal growth in YPD pH 7.8 as reference condition. In **(B)**, cells were grown in different culture media, mechanically broken, centrifuged to pellet cell debris and the cell homogenate used to quantify the total activity of α-mannosidase using the substrate 4-umbellipheryl-α-D-mannopyranoside. In **(C)**, cells were subjected to the same treatment as in **(B)**, but α-glucosidase activity using the substrate 4-umbellipheryl-α-D-glucopyranoside in absence or presence of 10 μM castanospermine was determined. ^∗^*P* < 0.05, when compared with untreated cells grown in YPD pH 7.8. ‡*P* < 0.05, when compared with untreated cells. ^†^Defined as nmoles of 4-methylumbellyferone min^-1^ total protein^-1^.

To demonstrate that the constitutive and stable expression of this gene could be useful for data normalization, during expression quantification of a target gene, we determined the relative quantification of the encoding genes for mannosyl-oligosaccharide glucosidase (Lopes-Bezerra et al., 2015), α-glucosidase II (Robledo-Ortiz et al., 2012) and mannosyl-oligosaccharide α-1,2-mannosidase (Lopes-Bezerra et al., 2015) by calculating 2^-ΔΔC_*t*_^ (Livak and Schmittgen, 2001). The endogenous control gene was the one encoding for the ribosomal protein L6, and as the reference condition the growth in YPD broth, pH 7.8. The expression of the three genes was similar when cells were grown in PDB or YPD, pH 4.5, when compared to the reference condition, i.e., cellular growth in YPD, pH 7.8 (**Figure 5A**). However, a significant increment in the expression of these three genes was observed when cells were grown in either Vogel medium or BHI (**Figure 5A**). Accordingly, when α-mannosidase activity was measured in cells grown under the same experimental conditions, this was significantly increased in cells grown in Vogel medium or BHI; while the enzyme activity from cells grown in PDB, YPD, pH 4.5, or pH 7.8, was significantly lower and similar across the three-growing conditions (**Figure 5B**). When a similar approach was used to quantify the α-glucosidase activity, again, only cells growing in Vogel medium or BHI had higher enzyme activity (**Figure 5C**). Since the approach to measure the α-glucosidase activity cannot distinguish between the mannosyl-oligosaccharide glucosidase and α-glucosidase II (Robledo-Ortiz et al., 2012; Lopes-Bezerra et al., 2015), we also quantified the enzyme activity in presence of castanospermine, an inhibitor with strong preference toward the mannosyl-oligosaccharide glucosidase (Mora-Montes et al., 2009; Lopes-Bezerra et al., 2015). Upon incubation with castanospermine, the enzyme activity was reduced in about 50% in all the growth conditions (**Figure 5C**), indicating both the mannosyl-oligosaccharide glucosidase and α-glucosidase II activities are being detected, and the changes in the enzyme levels, related to different growth conditions, are associated to changes in both enzyme activities. These data suggest the gene encoding for the ribosomal protein L6 was useful as endogenous control during data normalization.

**FIGURE 6:**
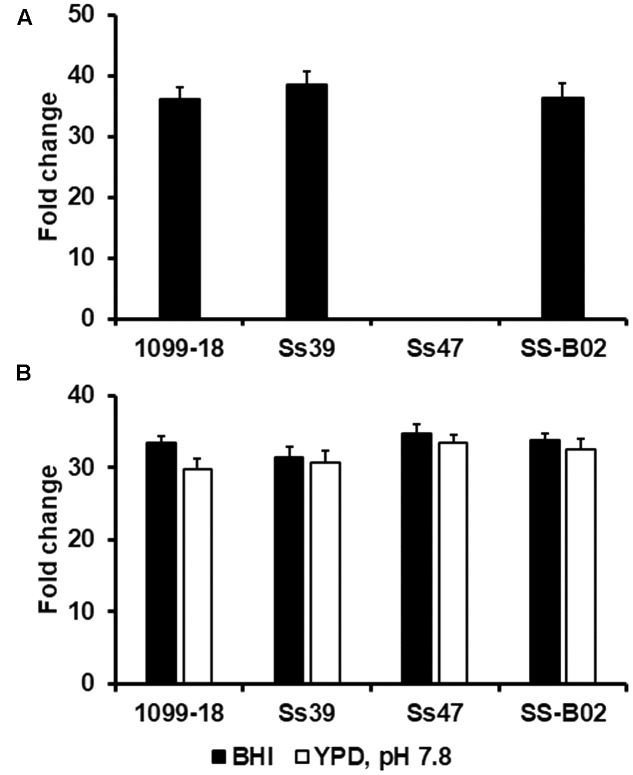
Expression of the gene encoding for β-fructofuranosidase activity and the hybrid histidine kinase DRK1 in different strains of *S. schenckii sensu stricto*. In **(A)**, cells were grown YNB added with 3% sucrose and were incubated at 28°C per 72 h. Total RNA was extracted, cDNA synthesized with oligo(dT) primer (20 mer) and the gene expression was quantified by RT-qPCR using specific primers (see **Table 1**). The reference condition to calculate the fold change was cells grown in YNB added with 3% glucose and the encoding gene for the ribosomal protein L6 was used as endogenous control. In **(B)**, cells were grown at 37°C in either BHI or YPD, pH 7.8, to induce yeast-like cell formation. Total RNA was extracted, cDNA synthesized with oligo(dT) primer (20 mer) and the gene expression was quantified by RT-qPCR using specific primers (see **Table 1**). The reference condition to calculate the fold change was cells grown at 28°C in BHI or YPD, pH 4.5, respectively. In both cases, the encoding gene for the ribosomal protein L6 was used as endogenous control. One-way ANOVA was used to establish statistical significance. In both panels, *P*-value was for all the strains > 0.05.

Next, to continue with this validation, conidia were grown in YNB added with either 3% glucose or 3% sucrose and were incubated at 28°C per 72 h. Using the growth in glucose as reference condition, and the encoding gene for the ribosomal protein L6 as endogenous control, we observed a 36.2 ± 1.9-fold increase in the expression of the encoding gene for β-fructofuranosidase activity when cells were grown in sucrose as a carbon source (**Figure 6A**). This data is similar to the inducible expression of *SUC2*, the putative functional ortholog from *Saccharomyces cerevisiae* (Lutfiyya and Johnston, 1996). The other fungal strains analyzed showed a similar profile, with exception of strain Ss47, which did not show gene expression in the media containing either glucose or sucrose (**Figure 6A**). PCR reactions were set to amplify the β-fructofuranosidase ORF from all the tested strains and this could not be amplified from strain Ss47 (data not shown). The sequencing of this region of the genome showed the β-fructofuranosidase ORF is absent from the genome of strain Ss47, which is likely to explain the lack of gene expression in this strain.

Finally, the hybrid histidine kinase DRK1 has been suggested to be involved in the *S. schenckii* dimorphism, and its expression is highly upregulated in yeast-like cells (Hou et al., 2013). To further demonstrate the gene encoding for the ribosomal protein L6 is a good candidate for data normalization in RT-qPCR, we measured the expression of the gene encoding DRK1 in yeast-like cells grown in BHI at 37°C, using as reference condition the fungal growth in the same culture medium at 28°C. Under these conditions, there was a 33.4 ± 0.9-fold increase in the expression of the gene encoding for DRK1, when compared to the reference condition, and was also observed in the other strains analyzed (**Figure 6B**). Similarly, when the experiment was conducted with yeast-like cells grown in YPD, pH 7.8, there was a 29.8 ± 1.5-fold increase in gene expression, when compared to cells grown in YPD, pH 4.5, at 28°C (**Figure 6B**).

## Discussion

*S. schenckii* and the closely related species that are responsible of sporotrichosis are getting more attention nowadays because the versatility of the clinical manifestations of the disease, the outbreaks transmitted by felines, and the interest of searching for new therapeutic alternatives to treat the infection (Lopez-Romero et al., 2011; Mora-Montes et al., 2015). This has led us to the genome sequencing of these organisms (Teixeira et al., 2014; D’Alessandro et al., 2016; Huang et al., 2016), and the standardization of molecular tools for gene manipulation (Rodriguez-Caban et al., 2011; Zhang et al., 2011). Gene silencing, disruption, edition or over expression are usually accompanied by analysis of gene expression, which rely on data normalization using endogenous genes with stable and constitutive expression across different growth conditions. Previous works in *S. schenckii* have used the gene encoding for β-tubulin (Hu et al., 2015), ribosomal protein L34 (Teixeira et al., 2015), and 18S ribosomal RNA (de Jesús-Berríos and Rodríguez-del Valle, 2002; Hou et al., 2013, 2014; Zhang et al., 2013). However, the expression of these genes is assumed to be stable and constitutive. Here, we experimentally tested the expression of these and other genes used for data normalization during expression analysis in fungal cells. Despite the 18S ribosomal RNA is widely used, it has the disadvantage that lacks polyadenylation and therefore, cDNA synthesis has to be performed with a specific reverse primer, precluding the use of this cDNA to amplify the target gene, i.e., the cDNA of the gene of interest has to be synthesized with a specific primer, which adds an additional step to the methodology, with potentially negative effects during the quantification reaction. Furthermore, the use of cDNA synthesized with the oligo(dT) primer (20 mer) has the advantage that can be used for RT-qPCR reactions targeting any gene capable of polyadenylating transcripts; and reduce the effects related with the efficiency of the retro transcriptase, which can have different processivity depending on the primer used (Zhang and Byrne, 1999).

Even though we aimed to test the expression of the selected genes in the three morphologies of *S. schenckii*, we failed to have reproducible data with conidia. A possible explanation for this observation would be the high increment of RNases in this cell morphology, or alternatively, the presence of increased amounts of molecules that quench the fluorescence dye. Since conidia are regarded as dormant structures with minimal or null transcription (Leng et al., 2008), we did not seek to establish the expression of the selected genes in this morphology. Nonetheless, it remains to be demonstrated whether the gene encoding for the ribosomal protein L6 has an expression comparable to that showed in both hyphae and yeast-like cells.

The gene encoding for the ribosomal protein L6 demonstrated to have stable and constitutive expression in both yeast-like cells and hyphae, and the medium composition had no influence in the gene expression. Moreover, this expression was constant during the interaction with host cells in both *in vivo* and *ex vivo* conditions, strengthening the hypothesis that can be used as an internal control for data normalization during analysis of gene expression. Most importantly, the expression of this gene did not show significant variations when fungal strains with different virulence were used in the *in vivo* and *ex vivo* interactions, suggesting that our observation about the stable and constitutive expression can be associated to the species rather than the strain used in this study. As a proof of concept, we demonstrated a strong correlation between the gene expression, calculated using the gene encoding for the ribosomal protein L6 as internal control, and the enzyme activity of both α-mannosidase and α-glucosidases. Despite there is more than one gene encoding for endoplasmic reticulum (ER) α-mannosidase-like proteins (Lopes-Bezerra et al., 2015), the mannosyl-oligosaccharide α-1,2-mannosidase is the main enzyme activity sensed with the substrate 4-methylumbellyferyl-α-D-mannopyranoside (Mora-Montes et al., 2004), thus it is likely to associate the enzyme activity quantified with the expression levels of this gene. Similarly, it has been demonstrated that 4-methylumbellyferyl-α-D-glucopyranoside is metabolized by ER glycosidases, in particular by mannosyl-oligosaccharide glucosidase and α-glucosidase II (Mora-Montes et al., 2009; Frade-Perez et al., 2010; Robledo-Ortiz et al., 2012), and the presence of castanospermine bias the system to quantify the enzyme activity associated to α-glucosidase II (Mora-Montes et al., 2009; Robledo-Ortiz et al., 2012; Lopes-Bezerra et al., 2015). Thus, the total quantification of the enzyme activity and the use of castanospermine allowed to confirm the correlation between gene expression and enzyme activity. The modulation of the expression of these genes has not been reported for fungal cells; thus, to our knowledge, this study reports for first time that ER α-glycosidases suffer gene regulation depending on the nutrients available in the media. BHI, the richest medium tested was associated with the highest expression and enzyme activity of ER α-glycosidases. The nutrient availability rather that cell morphology is likely to account for this observation, since cells grown in BHI and YPD, pH 7.8, displayed yeast-like morphology, but different levels in gene expression and enzyme activity of these glycosidases. Moreover, it has been reported in *Candida albicans* that the carbon source available for assimilation strongly influences the elaboration of cell wall components, including *N*-linked mannans (Ene et al., 2012a,b; Hall, 2015). It remains to be established whether other genes involved in the synthesis of *N*-linked mannans undergo gene regulation in rich media, such as that reported here for ER α-glycosidases.

The increased expression of the gene encoding for β-fructofuranosidase supports the cell growth in sucrose, although this gene upregulation in *S. schenckii* was not as high as that reported in *S. cerevisiae*, which can be up to 200-fold increase in presence of this carbon source (Lutfiyya and Johnston, 1996). As a possible explanation for this, the stability of the enzyme could be different in these organisms, having a half-life longer in *S. schenckii*, and thus, this species does not require a strong gene upregulation like that found in *S. cerevisiae*. Alternatively, it could be possible that functional paralogs would be present in *S. schenckii* and sugar metabolism depends on a gene family encoding proteins with β-fructofuranosidase activity. Once again, further experiments are required to provide a proper explanation for this apparent modest expression of the gene encoding for β-fructofuranosidase.

Finally, it is noteworthy to mention that the gene encoding for the ribosomal protein L6 could have changes in the expression levels in conditions not tested in this work, or in other species of the *Sporothrix* genus; therefore, the expression stability has to be evaluated before used as control in other experimental conditions or species. Though, this gene is currently being used by our group to quantify gene expression in *S. brasiliensis*, with results comparable to those reported here for *S. schenckii sensu stricto* (our unpublished data).

## Conclusion

We reported here that the gene encoding for the ribosomal protein L6 has stable and constitutive expression in different cell morphologies, growth conditions and during *in vivo* and *ex vivo* interaction with host cells. Therefore, it can be used as an internal control for data normalization during analysis of gene expression in *S. schenckii*.

## Author Contributions

ET-E, PP-N, and HM-M conceived the study; ET-E, JM-A, DC-G, and NH performed the experiments; ET-E, AF-M, PP-N, and HM-M analyzed the data; HM-M drafted the paper; ET-E, JM-A, DC-G, NH, AF-M, PP-N, and HM-M approved the final version of the manuscript.

## Conflict of Interest Statement

The authors declare that the research was conducted in the absence of any commercial or financial relationships that could be construed as a potential conflict of interest. The reviewer AR and handling Editor declared their shared affiliation.
